# The Durative Use of Suspension Cells and Callus for Volatile Oil by Comparative with Seeds and Fruits in *Capparis spinosa* L

**DOI:** 10.1371/journal.pone.0113668

**Published:** 2014-11-25

**Authors:** Yongtai Yin, Yuchi He, Wei Liu, Lu Gan, Chunhua Fu, Haibo Jia, Maoteng Li

**Affiliations:** 1 College of Life Science and Technology, Huazhong University of Science and Technology, Wuhan, 430074, P. R. China; 2 Key Laboratory of Molecular Biology, Physics of Ministry of Education, Huazhong University of Science and Technology, Wuhan, 430074, P. R. China; 3 Hubei Collaborative Innovation Center for Green Transformation of Bio-Resources, Faculty of Life Science, Hubei University, Wuhan, 430062, P. R. China; INRA, France

## Abstract

*Capparis spinosa* is one of the most important eremophytes among the medicinal plants, and continued destruction of these plants poses a major threat to species survival. The development of methods to extract compounds, especially those of medicinal value, without harvesting the whole plant is an issue of considerable socioeconomic importance. On the basis of an established system for culture of suspension cells and callus *in vitro*, Gas Chromatograph-Mass Spectrometer (GC-MS) was used for the volatile oil composition analyzing in seed, fruit, suspension cells and callus. Fatty acids were the major component, and the highest content of alkanes was detected in seed, with <1.0% in suspension cells and callus. Esters, olefins and heterocyclic compounds were significantly higher in fruit than in the other materials. The content of acid esters in the suspension cells and callus was significantly higher than in seed and fruit. This indicated that the suspension cells and callus could be helpful for increasing the value of volatile oil and replacing seeds and fruit partially as a source of some compounds of the volatile oil and may also produce some new medical compounds. The above results give valuable information for sustainable use of *C. spinosa* and provide a foundation for use of the *C. spinosa* suspension cells and callus as an ongoing medical resource.

## Introduction

The caper bush (*Capparis spinosa* L.) is a typical eremophyte, belonging to the Capparidaceae family that includes more than 250 species worldwide, and originates from tropical regions [1]. *Capparis spinosa* can utilize groundwater resources for the highly developed of the root and xylem systems, and is extensively distributed in drought-, cold- and heat-stressed environments; e.g. Mediterranean countries and Xinjiang and Gansu Provinces in China [2], [3]. *C. spinosa* is one of the most important medicinal plants and provides key resources for raw materials for the pharmaceutical, aromatic and food industries [4], [5]. There are many reports concerning the volatile oil, alkaloids, flavonoids, terpenes and mustard oil glycosides in *C. spinosa*. These constituents have antibiotic, anti-inflammatory, anti-oxidative and anti-hypertensive effects; they lower blood sugar and blood fat and are used for diuresis and treatment of arthritis and rheumatism [6]–[10]. As well as medical uses, all parts of *C. spinosa* have important nutritional value – e.g. aluminum, phosphorus, sodium, magnesium, iron and calcium – and the concentrations of heavy metals are below permissible safety limits [11].

Fruit and seed are important parts of *C. spinosa* in regard to quality and quantity of medically important components. The ripe fruit is rich in proteins, lipids, carbohydrates, vitamins and minerals. The ethanolic extract of *C. spinosa* fruit has protective activity against oxidative stress and interruption of the ROS-ERK1/2-Ha-Ras signal loop in systemic sclerosis, signifying its potential protective effect against skin sclerosis [12]. Both the oil and protein fractions can be extracted from *C. spinosa* seed and 145 compounds can be extracted from the volatile oil, with the major constituents being aldehydes, esters and sulfur-containing compounds [13]. The chemical composition of the oil shows that it contains fatty acids, tocopherols and sterols as well as glucosinolates [9]. Fatty acids, especially polyunsaturated fatty acids may affect cellular functions such as membrane-bound enzymes [14], transport systems [15] and receptors [16]. Various biochemical compounds, such as alkaloids, phenols and sterols might also have important medical value [17]. A novel dimeric 62-kDa lectin that can inhibit HIV-1 reverse transcriptase and proliferation of both hepatoma HepG2 and breast cancer MCF-7 cells was purified from *C. spinosa* seed [18]. These results emphasize the richness of sterols in the volatile oil of *C. spinosa* seed, and are quantitatively the most important class of minor components [19].

Currently, most *C. spinosa* volatile oil is isolated from different parts of the naturally growing plant, and the continued destruction of plants is a major threat to this species. The development of methods to extract compounds, especially those of medicinal value, without harvesting the whole plant is an issue of considerable socioeconomic importance. These factors have generated considerable interest in the use of plant cell culture technology for the production of pharmaceuticals [20]–[22]. Plant cell cultures have been used for plant propagation and to investigate physiological, biochemical and molecular aspects of various cellular functions [23]–[25]. Both the suspension cells and callus respond directly to physiological and biochemical factors and could be good models for studying potentially useful medical components. However, the development and commercialization of *C. spinosa* by bio-industries depends upon the availability of facilities and information concerning upstream and downstream bio-processing, extraction, purification and marketing of the industrial potential of *C. spinosa* [9]. In our previous work, we established a system for culture of the suspension cells and callus *in vitro* [25], [26].

In this paper, the volatile oil compounds were measured by gas chromatography-mass spectrometry (GC-MS) in fruit, seed, suspension cells and callus of *C. spinosa*. Detailed analysis of the volatile oil of these four kinds of materials would give valuable information for sustainable use of *C. spinosa* and provide a credible foundation for use of suspension cells and callus, instead of whole plants, as a continuously available resource.

## Results

### Composition of volatile oil extracted from different materials

**Figure 1:**
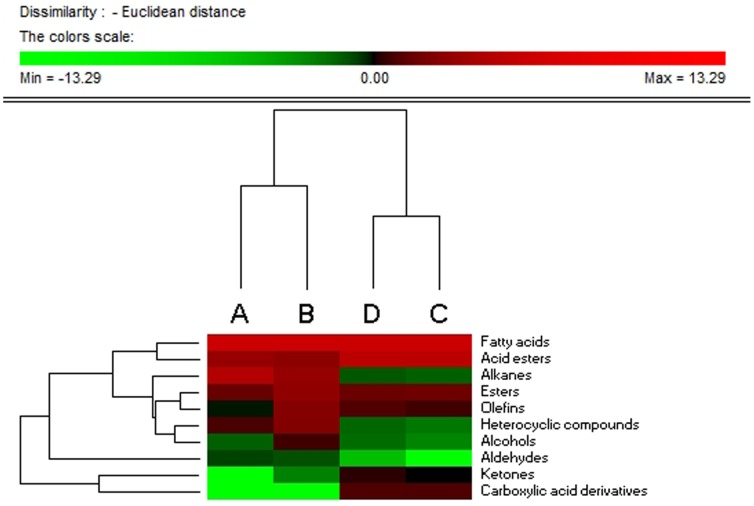
Hierarchical analysis of the total content of different groups in volatile oil in four kinds of materials. A, B, C and D represent the seeds, fruit, suspension cell and callus. Colors in the heatmap mean the fold change in according to the above, red and green represent the higher and lower levels, respectively.

The major components of the volatile oils extracted from *C. spinosa* seed, fruit, suspension cells and callus were fatty acids, alkanes, acid esters, esters and heterocyclic compounds (Table 1). The results indicated that different types of plant materials had quantitative and qualitative differences in the volatile oil (Table 1 and Fig. 1). This indicated that fatty acids were the major component (>60%) of the volatile oil in the four types of materials and the contents of the other components were quite varied. Alkanes were the second most abundant component in seed (21.21%), but were much less abundant in suspension cells and callus (<1.0%). The content of esters, olefins and heterocyclic compounds in fruit were 8.22, 5.05 and 4.95%, respectively, which were significantly higher than in the other three materials. The amounts of acid esters in suspension cells (28.39%) and callus (27.95%) were significantly higher than in seed and fruit. There were small amounts of carboxylic acid derivatives in the suspension cells and callus, and other compounds were identified in the extracts (Table 1). There were small amounts of alcohols, aldehydes and ketones in the volatile oils, in general, but no ketones in seed.

**Table 1:** The content of different composition of the volatile oil extracted from four kinds of materials.

	Content (%)			
	Seed	Fruit	Suspension cell	Callus
Fatty acids	62.96±1.21	63.75±2.27	61.11±1.78	60.55±1.31
Alkanes	21.21±0.99	7.98±0.74	0.41±0.01	0.47±0.03
Acid esters	8.25±0.24	6.21±0.69	28.39±0.15	27.95±1.11
Esters	2.83±0.14	7.21±0.83	3.13±0.07	2.88±0.33
Heterocyclic compounds	1.70±0.10	4.89±0.21	0.26±0.01	0.37±0.07
Olefins	0.96±0.06	5.09±0.23	1.50±0.03	1.90±0.20
Aldehydes	0.63±0.03	0.53±0.02	0.00	0.03±0.01
Alcohols	0.42±0.08	1.53±0.96	0.19±0.03	0.33±0.04
Carboxylic acid derivatives	0.00	0.00	1.84±0.21	1.79±0.46
Ketones	0.00	0.21±0.22	1.00±0.08	1.22±0.13
Total	98.96±0.18	97.39±2.32	97.83±1.45	97.49±0.51

**Figure 2:**
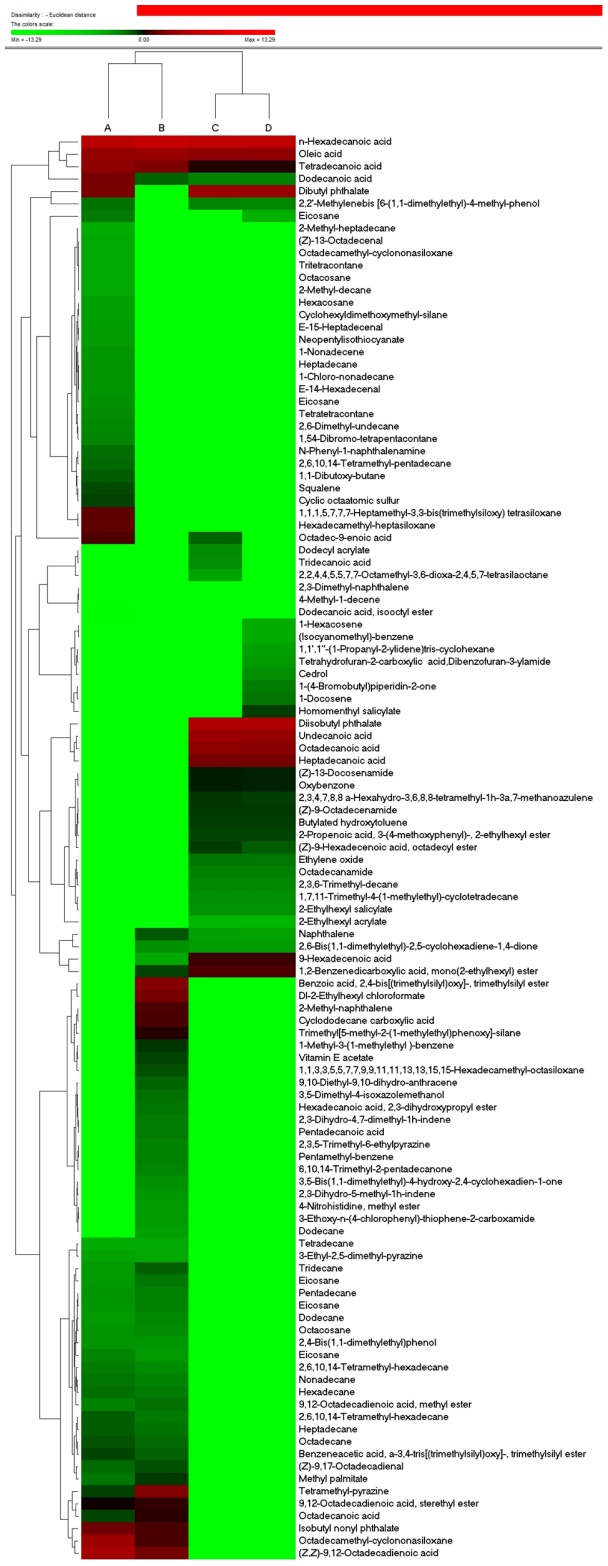
Hierarchical cluster analysis of all the detected compounds of volatile oil in four kinds of materials. A, B, C and D represent the seeds, fruit, suspension cell and callus. Colors in the heatmap mean the fold change in according to the above, red and green represent the higher and lower levels, respectively.

**Figure 3:**
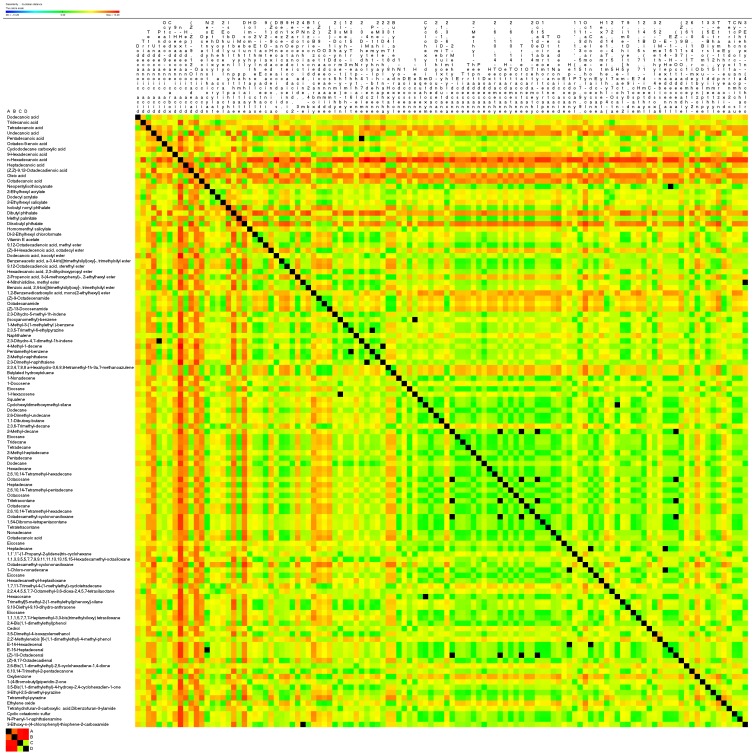
Heatmap of the correlation matrix of the volatile oil that detected in four kinds of materials. Positive correlations are shown in red; negative correlations in green; absence of correlation in black.

More than 110 compounds were identified: 13 fatty acids, 11 acid esters, 13 esters, 17 olefins, 40 alkanes, seven heterocyclics and some other compounds (Tables 2–4). Almost all the detected compounds showed some changes in levels in at least one of the four materials. Hierarchical cluster analysis showed that the suspension cells and callus had the most similar volatile oil profiles, while seed and fruit had the most differential profiles (Figs. 1 and 2). The callus and suspension cells had the most similar fatty acid, acid ester, ester and carboxylic acid profiles, while the fruit and seed had the most similar olefin, alkane, alcohol, aldehyde, ketone and heterocyclic profiles. Correlation analysis of volatile compounds (Fig. 3) was compared with hierarchical clustering, and also revealed that the highly positively correlated volatiles were generally in one group, e.g. n-hexadecanoic, oleic and tetradecanoic acids. The volatiles grouped in other clusters tended to be negatively correlated or had no significant correlations (Fig. 3). Detailed information for these compounds in the different materials follows.

**Table 2:** Fatty acid, acid esters, esters and carboxylic acid derivatives in four kinds of materials.

	Compound	Molecular formula	Molecular mass (kDa)	Content (%)			
				Seed	Fruit	Suspension cell	Callus
Fatty acid	Dodecanoic acid	C_12_H_24_O_2_	200.18	3.88±0.04	0.39±0.06	0.20±0.02	0.20±0.03
	Tridecanoic acid	C_13_H_26_O_2_	214.34	–	–	0.15±0.03	–
	Tetradecanoic acid	C_14_H_28_O_2_	228.21	6.60±0.06	4.31±0.15	1.13±0.31	1.15±0.05
	Undecanoic acid	C_11_H_22_O	186.29	–	–	8.74±0.34	7.76±0.71
	Pentadecanoic acid	C_15_H_30_O_2_	242.22	–	0.25±0.03	–	–
	Octadec-9-enoic acid	C_18_H_34_O_2_	282.26	1.83±0.15	–	0.38±0.13	–
	Cyclododecane carboxylic acid	C_13_H_24_O_2_	212.33	–	1.68±0.01	–	–
	9-Hexadecenoic acid	C_16_H_30_O_2_	254.22	–	0.07±0.00	1.47±0.22	1.46±0.21
	n-Hexadecanoic acid	C_16_H_32_O_2_	256.24	28.92±1.00	45.18±0.16	33.51±2.39	33.19±1.57
	Heptadecanoic acid	C_17_H_34_O_2_	270.46	–	–	3.79±0.15	3.76±0.27
	(Z,Z)-9,12-Octadecadienoic acid	C_18_H_32_O_2_	280.24	13.95±0.04	3.57±0.11	–	–
	Oleic acid	C_18_H_34_O_2_	282.26	7.78±0.22	8.79±0.16	6.55±0.57	6.99±0.11
	Octadecanoic acid	C_18_H_36_O_2_	284.27	–	–	6.09±1.20	6.04±0.14
Acid ester	Neopentylisothiocyanate	C_6_H_11_NS	281.46	0.10±0.01	–	–	–
	2-Ethylhexyl acrylate	C_11_H_20_O_2_	184.28	–	–	0.04±0.03	0.04±0.02
	Dodecyl acrylate	C_15_H_28_O_2_	240.38	–	–	0.16±0.02	–
	2-Ethylhexyl salicylate	C_15_H_22_O_3_	250.33	–	–	0.13±0.05	0.13±0.03
	Isobutyl nonyl phthalate	C_22_H_34_O_4_	362.25	3.40±0.22	1.73±0.05	–	–
	Dibutyl phthalate	C_16_H_22_O_4_	278.15	3.98±0.02	–	9.50±0.65	9.41±0.55
	Methyl palmitate	C_17_H_34_O_2_	270.26	0.26±0.04	0.72±0.03	–	–
	Diisobutyl phthalate	C_16_H_22_O_4_	278.34	–	–	18.56±0.55	17.68±1.63
	Homomenthyl salicylate	C_16_H_22_O_3_	262.34	–	–	–	0.69±0.00
	Dl-2-Ethylhexyl chloroformate	C_9_H_17_ClO_2_	192.09	–	3.79±0.12	–	–
	Vitamin E acetate	C_31_H_52_O_3_	472.74	–	0.63±0.00	–	–
Ester	9,12-Octadecadienoic acid, methyl ester	C_19_H_34_O_2_	294.26	0.20±0.02	0.30±0.05	–	–
	(Z)-9-Hexadecenoic acid, octadecyl ester	C_34_H_66_O_2_	506.89	–	–	0.68±0.01	0.45±0.06
	Dodecanoic acid, isooctyl ester	C_20_H_40_O_2_	312.30	1.00±0.02	–	–	–
	Benzeneacetic acid, α-3,4-tris[(trimethylsilyl)oxy]-, trimethylsilyl ester	C_20_H_40_O_5_Si_4_	472.19	0.61±0.04	0.40±0.08	–	–
	9,12-Octadecadienoic acid, sterethyl ester	–	–	1.02±0.02	1.32±0.04	–	–
	Hexadecanoic acid, 2,3-dihydroxypropyl ester	C_19_H_38_O_4_	330.50	–	0.27±0.04	–	–
	2-Propenoic acid, 3-(4-methoxyphenyl)-, 2-ethylhexyl ester	C_18_H_26_O_3_	290.40	–	–	0.61±0.06	0.61±0.14
	4-Nitrohistidine, methyl ester	C_7_H_10_N_4_O_4_	214.18	–	0.1±0.01	–	–
	Benzoic acid, 2,4-bis[(trimethylsilyl)oxy]-, trimethylsilyl ester	C_16_H_30_O_4_Si_3_	370.66	–	5.19±0.14	–	–
	1,2-Benzenedicarboxylic acid, mono(2-ethylhexyl) ester	C_16_H_22_O_4_	278.15	–	0.64±0.02	1.84±0.13	1.82±0.51
Carboxylic acid derivative	(Z)-9-Octadecenamide	C_18_H_35_NO	281.48	–	–	0.71±0.09	0.70±0.23
	Octadecanamide	C_18_H_37_NO	283.49	–	–	0.21±0.01	0.21±0.03
	(Z)-13-Docosenamide	C_22_H_43_NO	337.58	–	–	0.92±0.13	0.88±0.21

– Represents compounds not detected.

**Table 3:** Olefin and alkanes in four different plant materials.

	Compound	Molecular formula	Molecular mass (kDa)	Content (%)			
				Seed	Fruit	Suspension cell	Callus
Olefin	2,3-Dihydro-5-methyl-1h-indene	C_10_H_12_	132.09	–	0.12±0.01	–	–
	(Isocyanomethyl)-benzene	C_8_H_7_N	161.2	–	–	–	0.06±0.03
	1-Methyl-3-(1-methylethyl)-benzene	C_10_H_14_	134.22	–	0.73±0.05	–	–
	2,3,5-Trimethyl-6-ethylpyrazine	C_9_H_14_N_2_	150.12	–	0.21±0.03	–	–
	Naphthalene	C_10_H_8_	128.06	–	0.48±0.06	0.09±0.02	0.08±0.05
	2,3-Dihydro-4,7-dimethyl-1h-indene	C_11_H_14_	146.11	–	0.25±0.01	–	–
	4-Methyl-1-decene	C_11_H_22_	154.17	–	–	–	–
	Pentamethyl-benzene	C_11_H_16_	148.24	–	0.21±0.02	–	–
	2-Methyl-naphthalene	C_11_H_10_	142.08	–	1.79±0.01	–	–
	2,3-Dimethyl-naphthalene	C_12_H_12_	156.22	–	–	–	–
	2,3,4,7,8,8 a-Hexahydro-3,6,8,8-tetramethyl-1h-3a,7-methanoazulene	C_15_H_24_	204.35	–	–	0.73±0.13	0.68±0.15
	Butylated hydroxytoluene	C_15_H_24_O	220.34	–	–	0.68±0.13	0.68±0.03
	1-Nonadecene	C_19_H_38_	266.30	0.12±0.04	–	–	–
	1-Docosene	C_22_H_44_	308.58	–	–		0.28±0.06
	Eicosane	C_20_H_42_	228.55	0.19±0.03	0.11±0.01	–	–
	1-Hexacosene	C_26_H_52_	364.69	–	–	–	0.06±0.01
	Squalene	C_30_H_50_	410.72	0.57±0.02	–	–	–
Alkanes	Cyclohexyldimethoxymethyl-silane	C_9_H_20_O_2_Si	188.12	0.09±0.03	–	–	–
	Dodecane	C_12_H_26_	170.20	0.11±0.02	0.18±0.01	–	–
	2,6-Dimethyl-undecane	C_13_H_28_	184.22	0.18±0.04	–	–	–
	1,1-Dibutoxy-butane	C_12_H_26_O_2_	202.33	0.45±0.02	–	–	–
	2,3,6-Trimethyl-decane	C_13_H_28_	184.36	–	–	0.18±0.01	0.18±0.04
	2-Methyl-decane	C_11_H_24_	156.19	0.07±0.00	–	–	–
	Eicosane	C_20_H_42_	282.33	0.11 ±0.05	0.26±0.01	–	–
	Tridecane	C_13_H_28_	184.22	0.11±0.01	0.42±0.02	–	–
	Tetradecane	C_14_H_30_	198.24	0.06±0.00	0.07±0.01	–	–
	2-Methyl-heptadecane	C_18_H_38_	254.49	0.06±0.02	–	–	–
	Pentadecane	C_15_H_32_	212.25	0.13±0.11	0.21±0.03	–	–
	Dodecane	C_12_H_26_	170.20	–	0.09±0.01	–	–
	Hexadecane	C_16_H_34_	226.27	0.30±0.03	0.24±0.01	–	–
	2,6,10,14-Tetramethyl-hexadecane	C_20_H_42_	282.33	0.23±0.02	0.16 ±0.01	–	–
	Octacosane	C_28_H_58_	394.45	0.07±0.02	–	–	–
	Heptadecane	C_17_H_36_	240.28	0.43±0.01	0.29±0.08	–	–
	2,6,10,14-Tetramethyl-pentadecane	C_19_H_40_	268.31	0.34±0.02	–	–	–
	Octacosane	C_28_H_58_	394.76	0.13±0.01	0.16 ±0.02	–	–
	Tritetracontane	C_43_H_88_	604.69	0.07±0.02	–	–	–
	Octadecane	C_18_H_38_	254.30	0.52±0.04	0.35±0.04	–	–
	2,6,10,14-Tetramethyl-hexadecane	C_20_H_42_	282.33	0.43±0.03	0.24 ±0.05	–	–
	Octadecamethyl-cyclononasiloxane	C_18_H_54_O_9_Si_9_	667.39	0.07±0.01	–	–	–
	1,54-Dibromo-tetrapentacontane	C_54_H_108_Br_2_	914.68	0.19±0.02	–	–	–
	Tetratetracontane	C_44_H_90_	618.70	0.16±0.01	–	–	–
	Nonadecane	C_19_H_40_	268.52	0.27±0.02	0.20±0.02	–	–
	Octadecanoic acid	C_18_H_36_O_2_	284.27	0.61±0.02	1.22±0.1	–	–
	Eicosane	C_20_H_42_	282.33	0.25±0.02	–	–	0.05±0.00
	Heptadecane	C_17_H_36_	240.28	0.13±0.02	–	–	–
	1,1′,1″-(1-Propanyl-2-ylidene)tris-cyclohexane	C_21_H_38_	290.53	–	–	–	0.09±0.03
	1,1,3,3,5,5,7,7,9,9,11,11,13,13,15,15-Hexadecamethyl-octasiloxane	C_16_H_50_O_7_Si_8_	579.25	–	0.53±0.03	–	–
	Octadecamethyl-cyclononasiloxane	C_18_H_54_O_8_Si_9_	667.39	10.45±0.60	1.70 ±0.14	–	–
	1-Chloro-nonadecane	C_19_H_39_Cl	302.27	0.13±0.02	–	–	–
	Eicosane	C_20_H_42_	282.55	0.12±0.07	0.20±0.10	–	–
	Hexadecamethyl-heptasiloxane	C_16_H_48_O_6_Si_7_	533.15	2.42±0.41	–	–	–
	1,7,11-Trimethyl-4-(1-methylethyl)-cyclotetradecane	C_20_H_40_	280.53	–	–	0.15±0.01	0.15±0.05
	2,2,4,4,5,5,7,7-Octamethyl-3,6-dioxa-2,4,5,7-tetrasilaoctane	C_10_H_30_O_2_Si_4_	294.69	–	–	0.08±0.02	–
	Hexacosane	C_26_H_54_	366.71	0.09±0.04	–	–	–
	Trimethyl[5-methyl-2-(1-methylethyl)phenoxy]-silane	C_13_H_22_OSi	222.4	–	1.19±0.18	–	–
	9,10-Diethyl-9,10-dihydro-anthracene	C_18_H_20_	236.35	–	0.39±0.05	–	–
	Eicosane	C_20_H_42_	282.55	0.14±0.02	–	–	–
	1,1,1,5,7,7,7-Heptamethyl-3,3-bis(trimethylsiloxy) tetrasiloxane	C_13_H_40_O_5_Si_6_	444.97	2.29±0.05	–	–	–

– Represents compounds not detected.

**Table 4:** Alcohols, aldehydes, ketones, heterocyclic compounds composition comparison among the different four materials.

	Compound	Molecular formula	Molecular mass (kDa)	Content (%)			
				Seed	Fruit	Suspension cell	Callus
Alcohol	2,4-Bis(1,1-dimethylethyl)phenol	C_14_H_22_O	206.17	0.13±0.02	0.12±0.01	–	–
	Cedrol	C_15_H_26_O	222.34	–	–	–	0.15±0.06
	3,5-Dimethyl-4-isoxazolemethanol	C_6_H_9_NO_2_	127.12	–	0.30±0.01	–	–
	2,2′-Methylenebis [6-(1,1-dimethylethyl)-4-methyl-phenol	C_23_H_32_O_2_	340.24	0.29±0.07	–	0.19±0.03	0.18±0.03
Aldehyde	12-Octadecenal	C_18_H_34_O	266.46	–	–	–	0.03±0.01
	E-14-Hexadecenal	C_16_H_30_O	238.23	0.13±0.02	–	–	–
	E-15-Heptadecenal	C_17_H_32_O	252.25	0.10±0.01	–	–	–
	(Z)-13-Octadecenal	C_18_H_34_O	266.46	0.07±0.01	–	–	–
	(Z)-9,17-Octadecadienal	C_18_H_32_O	264.25	0.33±0.02	0.54±0.02	–	–
Ketone	2,6-Bis(1,1-dimethylethyl)-2,5-cyclohexadiene-1,4-dione	C_14_H_20_O_2_	220.15	–	0.14±0.00	0.10±0.02	0.10±0.00
	6,10,14-Trimethyl-2-pentadecanone	C_18_H_36_O	268.48	–	0.19±0.00	–	–
	Oxybenzone	C_14_H_12_O_3_	228.24	–	–	0.90±0.08	0.89±0.14
	1-(4-Bromobutyl)piperidin-2-one	C_9_H_16_BrNO	234.13	–	–	–	0.23±0.02
	3,5-Bis(1,1-dimethylethyl)-4-hydroxy-2,4-cyclohexadien-1-one	C_14_H_22_O_2_	222.32	–	0.14 ±0.03	–	–
Heterocyclic	3-Ethyl-2,5-dimethyl-pyrazine	C_8_H_12_N_2_	136.10	0.08±0.01	0.07±0.02	–	–
	Tetramethyl-pyrazine	C_8_H_12_N_2_	136.10	0.66±0.05	4.78±0.02	–	–
	Ethylene oxide	C_2_H_4_O	44.05	–	–	0.26±0.01	0.26±0.08
	Tetrahydrofuran-2-carboxylic	C_17_H_15_NO_3_	281.31	–	–	–	0.11±0.04
	acid,Dibenzofuran-3-ylamide						
	Cyclic octaatomic sulfur	S_8_	256.52	0.64±0.03	–	–	–
	N-Phenyl-1-naphthalenamine	C_16_H_13_N	219.10	0.32±0.03	–	–	–
	3-Ethoxy-n-(4-chlorophenyl)-thiophene-2-carboxamide	C_13_H_12_ClNO_2_S	281.76	–	0.10±0.01	–	–

– Represents compounds not detected.

### Fatty acids, acid esters, esters and carboxylic acid derivatives in four materials

Of the fatty acids in volatile oil extracted from the four materials (Table 2), the main components were n-hexadecanoic, oleic, tetradecanoic and dodecanoic acids. The content of tetradecanoic acid in seed and fruit was greater than in suspension cells and callus, and the content of dodecanoic acid varied greatly among the four materials: 3.88% in seed but very much less in the other three materials. There were six kinds of fatty acids in the volatile oil extracted from seed – the major components were n-hexadecanoic (28.92%), (Z,Z)-9,12-octadecadienoic (13.95%), oleic (7.78%), tetradecanoic (6.60%) and dodecanoic (3.88%) acids, together representing 61.13% of the total. Octadec-9-enoic acid was found only in seed. The primary fatty acids in fruit were n-hexadecanoic (45.18%), oleic (8.79%), tetradecanoic (4.31%) and (Z,Z)-9,12-octadecadienoic (3.57%) acids, together representing 61.85% of the total. Pentadecanoic and cyclododecanecarboxylic acids were found only in fruit and (Z,Z)-9,12-octadecadienoic acid was the only fatty acid found in seed and fruit; whereas n-hexadecanoic acid was found in all four materials, with up to 45.18% in fruit. Nine and eight kinds of fatty acids were observed in suspension cells and callus, respectively, with n-hexadecanoic, oleic, undecanoic, oleic and octadecanoic being the major fatty acids, and of similar content in both materials (Table 2). Undecanoic, octadecanoic and heptadecanoic acids were found in suspension cells and the callus, whereas tridecanoic acid was present only in suspension cells.

There were five different acid esters in seed, four in fruit and five in suspension cells and callus. The contents of dibutylphthalate and isobutylnonylphthalate in seed were relatively high with proportions of 3.98 and 3.40%, respectively. Isobutylnonylphthalate, methylpalmitate and DL-2-ethylhexylchloroformate were synthesized in seed and fruit – the content of isobutylnonylphthalate in seed was 3.40%, and was significantly lower at 1.73% in fruit; whereas neopentylisothiocyanate was present only in seed. Dibutylphthalate was present in seed, suspension cells and callus, with contents in suspension cells (9.50%) and callus (9.41%) greater than in seed (3.98%). Vitamin E acetate (0.63%) was a fruit-specific material. 2-Ethylhexylacrylate, 2-ethylhexylsalicylate and diisobutylphthalate were found only in suspension cells and callus. Diisobutylphthalate was abundant in suspension cells (18.56%) and callus (17.68%). Dodecyl acrylate was present only in suspension cells (0.16%) and homomethyl salicylate was callus specific (0.69%). No acid esters were present in fruit and callus. Other kinds of acid esters present in all four materials are given in Table 2.

Esters were present in small amounts in the volatile oil with four, eight, three and three kinds observed in seed, fruit, suspension cells and callus, respectively. Esters, including 9,12-octadecadienoic acid, sterethyl ester, benzeneacetic acid, α-3,4-tris[(trimethylsilyl)oxy]-, trimethylsilyl ester and 9,12-octadecadienoic acid were abundant in fruit, with yields up to 8.22%. The content of methyl esters was similar in seed and fruit. Dodecanoic acid and isooctyl ester were present only in seed. Benzoic acid, 2,4-bis[(trimethylsilyl)oxy]-trimethylsilyl ester (5.19%), hexadecanoic acid, 2,3-dihydroxypropyl ester (0.27%) and 4-nitrohistidine methyl ester (0.10%) were fruit specific. (Z)-9-Hexadecenoic acid, octadecyl ester, 2-propenoic acid and 3-(4-methoxyphenyl)-2-ethylhexyl ester were only present in suspension cells and callus at similar levels. The greatest contents of 1,2-benzenedicarboxylic acid and mono(2-ethylhexyl) ester were in suspension cells and callus, and were only in small amounts in fruit but absent from seed. There were no carboxylic acid derivatives in seed or fruit (Table 2). The contents of carboxylic acid derivatives were very low and similar in volatile oil extracted from suspension cells and callus, where the major components were (Z)-13-docosenamide, (Z)-9-octadecenamide and octadecanamide, respectively.

### Olefins and alkanes in four materials

Olefins accounted for only a small proportion of the volatile oils in the four materials (Table 3). There were four kinds of olefins in seed, but all at low levels; 4-methyl-1-decene, 1-nonadecene and squalene were present only in seed. In fruit, there were small amounts of nine kinds of olefins, including 1-methyl-3-(1-methylethyl)-benzene (0.73%), 2,3,5-trimethyl-6-ethylpyrazine (0.21%) and 2,3-dihydro-5-methyl-1h-indene (0.12%). There were only four olefin species in suspension cells, which included 2,3,4,7,8,8 a-hexahydro-3,6,8,8-tetramethyl-1h-3a,7-methanoazulene (0.68%), butylated hydroxytoluene (0.68%), naphthalene (0.09%) and squalene (0.05%) – with similar contents in callus to those in suspension cells. Naphthalene was present in fruit, suspension cells and callus; the greatest content was in fruit (0.48%) and with very small amounts in the other two samples. There were more olefin species (up to seven) in callus than in suspension cells, and 2,3,4,7,8,8a-hexahydro-3,6,8,8-tetramethyl-1h-3a,7-methanoazulene (0.68%) and butylated hydroxytoluene (0.68%) were present in relatively higher contents. Isocyanomethylbenzene, 1-docosene and 1-nonadecene were present exclusively in callus.

There were 30 kinds of alkanes in seeds, with a total content of 21.21%. Alkanes were also quantitatively the major component of fruit with yields up to 8.10%. However, alkanes were quantitatively only a minor proportion (<1.00%) in suspension cells and callus (Table 3). A total of 19 alkanes were seed-specific, including octadecamethyl-cyclononasiloxane (3.50%), hexadecamethyl-heptasiloxane (2.42%) and 1,1,1,5,7,7,7-heptamethyl-3,3-bis (trimethylsiloxy) tetrasiloxane (2.29%). There were 19 alkanes in fruit and octadecamethyl-cyclononasiloxane had the highest content (1.70%). Four were synthesized only in fruit, including trimethyl[5-methyl-2-(1-methylethyl)phenoxy]-silane (1.19%), 1,1,3,3,5,5,7,7,9,9,11,11,13,13,15,15-hexadecamethyl-octasiloxane (0.53%) 9,10-diethyl-9,10-dihydro-anthracene (0.39%) and dodecane (0.09%). In all, 14 alkanes were observed in both seed and fruit. Only three alkanes were synthesized in suspension cells and were also present in callus: 2,2,4,4,5,5,7,7-octamethyl-3,6-dioxa-2,4,5,7-tetrasilaoctane (0.08%), 2,3,6-trimethyl-decane (0.18%) and 1,7,11-trimethyl-4-(1-methylethyl)-cyclotetradecane (0.15%). There were four alkane species in callus, with 1,1′,1″-(1-propanyl-2-ylidene) tris-cyclohexane being unique.

### Alcohols, aldehydes, ketones and heterocyclics in four materials

Very few species of alcohol were present in any of the four materials (Table 4). There were two alcohols in seed, fruit and callus but only one in suspension cells. 2,4-Bis(1,1-dimethylethyl) phenol was present in similar amounts in both seed and fruit but was not present in suspension cells or callus; and 3,5-dimethyl-4-isoxazolemethanol was found only in fruit. Cedrol was found only in callus (0.15%) and 2,2′-methylenebis [6-(1,1-dimethylethyl)-4-methyl-phenol was present in small, similar amounts in suspension cells and callus, in slightly smaller amounts in seed and was not found in fruit. There were four kinds of aldehydes in seeds: e-14-hexadecenal (0.13%), e-15-heptadecenal (0.10%) and (Z)-13-octadecenal (0.07%) were present only in the seed; and (Z)-9,17-octadecadienal was present in both seed and fruit, with its content (0.54%) in fruit greater than in seed. There were very small amounts of 12-octadecenal (0.03%) in callus and none in the other materials. There was no aldehyde in suspension cells (Table 4).

There were three kinds of ketones in fruits, including 6,10,14-trimethyl-2-pentadecanone (0.19%), 3,5-bis(1,1-dimethylethyl)-4-hydroxy-2,4-cyclohexadien-1-one (0.14%) and 2,6-bis(1,1-dimethylethyl)-2,5-cyclohexadiene-1,4-dione (0.14%), which was also present in suspension cells and callus. No ketones were observed in seed. Oxybenzone, the most abundant ketone, was present at similar levels in suspension cells and callus but there was none in seed or fruit. A total of seven heterocyclic compounds were observed in the four materials: cyclic octaatomic sulfur and n-phenyl-1-naphthalenamine were only present in seed; whereas 3-ethoxy-N-(4-chlorophenyl)-thiophene-2-carboxamide and tetrahydrofuran-2-carboxylic acid dibenzofuran-3-ylamide were only in fruit and callus, respectively. Ethylene oxide was found in both suspension cells and callus and 3-ethyl-2,5-dimethyl-pyrazine and tetramethyl-pyrazine were in both seed and fruit.

## Discussion

*Capparis spinosa* is used as a Chinese traditional medicine for diuresis, lowering blood sugar and blood fat, as well as an arthritis and rheumatism treatment. Studies have revealed that many of its chemical constituents have antimicrobial, anti-oxidative, anti-inflammatory, immunomodulatory and/or antiviral properties [9], [10], [19]. In China, *C. spinosa* grows mainly in the dry sand of deserts and on the sunny slopes of low hills and is distributed widely in Xinjiang, but with limited total biomass. Volatile oil is abundant in seeds and the content of unsaturated fatty acids is very high. In general, the aerial parts of the plant have a high nutritional value [24], [25]. Environmental and geopolitical instability coupled with the rapid disappearance of the natural habitats of many medicinal plants makes it increasingly difficult to acquire large amounts of plant-derived compounds. Regeneration *in vitro* holds tremendous potential for production of high-quality, plant-based medicine [27]. This has prompted industry as well as academia to investigate cell culture as an alternative approach to the supply of raw materials for production of plant-based pharmaceuticals [22]. Most of the current valuable phytochemicals are products of plant secondary metabolism. Production of these secondary metabolites is possible via plant cell and tissue culture *in vitro* and this has made it possible to gradually replace some whole-plant cultivation as a source of useful secondary metabolites [20]–[22]. Recent developments in plant tissue culture techniques and processing have shown great promise and this *in vitro* approach could provide continuous and reliable sources of plant pharmaceuticals. The large-scale culture of plant cells from which these medical metabolites can be extracted will also contribute to the discovery of new medicines [22]. In previous studies, we established an *in vitro* culture system for production of suspension cells and callus of *C. spinosa* [24], [25], which provided the basis of the work described here.

A series of studies have reported the volatile oil content of different *C. spinosa* materials and the relevant applications [4], [5]. The chemical components of the volatile oil extracted from *C. spinosa* fruit are mainly unsaturated acids, esters and alkyl compounds [28]. Unsaturated acids account for 63.39%, esters for 6.84% and alkyl compounds for 4.23% of the volatile oil extracted from seeds [8]. The seeds of *C. spinosa* are a potential source of volatile oil that contains abundant unsaturated fatty acids, mainly oleic acid [5]. Those studies were mainly focused on the content of the volatile oil in fruit and seed, and the composition of the volatile oil of the suspension cells and callus of *C. spinosa* has not been reported. In this work, GC-MS was used to measure the content and composition of volatile oil in fruit, seed, suspension cells and callus of *C. spinosa*. Subsequently, the content and composition of the volatile oil were compared among these four materials. This information provides a credible foundation for using *C. spinosa* suspension cells and callus as a continuing medical resource in some cases.

The results showed the main component of volatile oil (especially fatty acids) was similar in seed, fruit, suspension cells and callus. Fatty acids were the major component, representing>60% of the volatile oil, with the richest species being oleic, tetradecanoic, dodecanoic and linoleic acids. Over 100 compounds were unequivocally identified both in the natural materials and materials cultured *in vitro*. The results indicated that some important fatty acids were only synthesized *in vitro* with high contents, e.g. undecanoic acid, and some were only synthesized *in vivo*, e.g. (Z,Z)-9,12-octadecadienoic acid was only observed in seed and fruit. Large amounts of acid esters were synthesized in suspension cells (28.39%) and callus (27.95%), and this was quantitatively the second main component after fatty acids, with contents significantly higher than in seed and fruit. A small amount of carboxylic acid derivatives were synthesized in suspension cells and callus. Although the total content volatile oil was same in the four types of materials, but the contents of the other components were quite varied. Some scientists have revealed that the glands in leaves and surface of fruit tissues are the site of storage and most likely biosynthesis of volatile compound [29], [30]. The hormonal composition of the culture media could alter the plant biomass capacity to produce secondary metabolites [31]. Therefore, the significant reduction in the production of monomeric compound in the seed, fruit, suspension cell and callus may be due to the lack of these glands and secretory channels in suspension cell and callus and the hormonal composition of the culture media. Therefore, the phenomenon offered the opportunity to fine-tune the type and concentration of hormone, either alone or in combination with nutrients in the culture medium to maximize the biomass yield and its secondary metabolite content.

Previous studies showed that *C. spinosa* has multiple functions in antibiotic, anti-inflammatory, anti-oxidative and anti-hypertensive effects, which were correlated with polyphenols, alkaloids, flavonoids, terpenes and mustard oil glycosides. Whether there are other groups responsible for the bio-functions mentioned above is not clear. In this study, oleic, tetradecanoic and dodecanoic acids were all found in the four materials in relatively greater quantities. Evidence from epidemiological studies suggested that a higher proportion of, especially oleic acid, in the diet is linked to reduced risk of coronary heart disease and is considered to be antithrombotic compared with saturated fatty acids. It is clear that antioxidants can reduce cancer, heart disease and other degenerative problems associated with aging [32]. It has also been reported that many fatty acids can act as antioxidants or prooxidants. Lauric, tridecanoic and myristic acids had been shown to have high antioxidant activities and to prevent cell death *in vitro* [33]. Analysis of components in the fatty acids group provided us a new perspective for understanding the therapeutic function of *C. spinosa*. In conclusion, different kinds of materials showed quantitative and qualitative differences in the volatile oil, and the *in vitro* or *in vivo* systems could be chosen according to the purpose. Culture factors could be regulated *in vitro* to obtain specific medicinal compositions. Thus, the *in vitro* culture system could be not only used as a pathway for the production of medical components such as certain special fatty acids, but also play an important role in discovery of new medicines through regulating the culture parameters – manipulation of physical aspects and nutritional elements in a culture is perhaps the most fundamental approach for optimization of culture productivity [20], [22]. For example, *in vitro* cell culture of *Taxus* spp. is one approach used to provide a stable supply of taxol and related taxane derivatives [34], [35]. Imperatorin was formed from *Angelica dahurica* formosana by cell suspension culture [36] and cryptotanshinone was produced from culture of *Salvia miltiorrhiza* Bunge callus [37]. Research into the chemical composition of volatile oil in the fruit, seed, suspension cells and callus of *C. spinosa* provided a theoretical basis for the comprehensive development and use of the resources of *C. spinosa* in medicine, food, cosmetics and other industries, i.e. a credible foundation for making use of *C. spinosa* suspension cells and callus as continuous medical resources instead of using whole plants. The results will be helpful in judging the nutritional and medical value and the use of Chinese *C. spinosa* in the future. The present results will lead to further insights into the biosynthetic pathways of volatiles in seed, fruit, suspension cells and callus of *C. spinosa* and will also supply information for the identification of genetic and environmental effects in volatile production [38], [39].

## Materials and Methods

### Experimental materials

Test materials of the fruit and seed of *C. spinosa* were collected from Bachu County (39.49N, 76.74E), Kashi, Xinjiang of China. No specific permissions were required for these locations/activities, the location is not privately-owned or protected in any way, the field studies did not involve endangered or protected species. For callus induction: the leaves and the stems of *C. spinosa* were cut into 0.5 cm×0.5 cm scraps and 1.0-cm long slices – these two kinds of explants were subcultured on MS medium supplemented with 1.5 mg/L 2,4-D (2,4-dichlorophenoxyacetic acid) and 3.0 mg/L 6-BA (6-benzylaminopurine) and 3% sucrose (pH 6.0), incubated in darkness for one week and culture then continued in the light. The MS medium with 1.5 mg/L 6-BA and 1.0 mg/L 2,4-D added was used for callus multiplication [24]. The fruit and mature seeds had been collected in perennial plant of *C. spinosa* after pollination for about 120 days. The Callus was initiated by leaf explants that cultured on MS medium that Murashige and Skoog reported [40] and collected after 8 weeks cultivation. The Suspension cells that grown in logarithmic growth was used volatile oil extraction.

The suspension cells were cultivated in liquid MS culture medium, which contained 2,4-D (1.0 mg/L) and 6-BA (1.5 mg/L). The media used for suspension cell culture had pH 5.8, the suspension cells was cultured at 25°C and the intensities of illumination were 25 µmol/(m^2^·s)for light (14 h) and dark (10 h) culture, respectively. Suspension cells in logarithmic growth phase were used for extraction of volatile oil [24].

### Extraction of volatile oil

The fruit, seed, suspension cells and callus of *C. spinosa* were washed and then dried in the shade, passed through a 60-mesh sieve, 25 g of materials accurately weighed and then placed in 1-L round bottom flask. Then 500 ml water was added by the determination of volatile oil of armor method, using the determination apparatus to extract the volatile oil for about 5–6 h, until no more droplets appeared. Separation of reservoir after standing, the aqueous phase was extracted three times with ether into the reservoir, then activated anhydrous sodium sulfate was used to dry it and finally a rotary evaporator used to remove the ether and produce a rich aroma of pale yellow oily substance, sealed, dark, cold standby. Supelco SPME (Solid Phase Micro Extraction) devices coated with polydimethylsiloxane (PDMS, 100 µm) were used to sample the headspace of 2 mL of extracted oil, which was inserted into a 5-mL glass septum vial and allowed to equilibrate for 20–30 min. After the equilibration, the fiber was exposed to the headspace for 50 min at 25°C, then withdrawn into a needle and transferred to the injection port on the GC-MS system [41]. The total number of analyses was 12 (three biological samples replicates for four varieties).

### Analysis of volatile oil components

A GC-MS 7890A equipped with an Agilent 5975C quadrupole mass spectrometer controlled by Enhanced ChemStation MSD ChemStation E.01.00.237 (Agilent, Santa Clara, CA) was used to analyze compounds in the extracted volatile oil. Gas chromatography conditions: column was a HP INNOWax (30 m×0.25 mm×0.25 µm), inlet temperature 200°C, column flow 1.0 mL/min, ion source temperature 200°C, injection volume 1 µL and split ratio 10:1. Temperature-programmed conditions: initial temperature 80°C, with 10°C/min rise to 150°C, maintained 1 min, then with 5°C/min rise to 200°C, maintained 3 min, then with 5°C/min rise to 250°C and the carrier gas was He [42]–[44]. The content was calculated by peak area normalization method.

### Gas chromatography identification results

Capillary gas chromatography was used to analyze the composition of volatile oil in the fruit, seed, suspension cells and callus of *C. spinosa*. The GC area normalization method was used for determination of the percentage content of each component. An Electron Impact (EI) mass spectral library with 220,460 spectra of 192,108 unique compounds, with identifications and usual chemical structures and MS/MS library with 14,802 spectra of 5,308 precursor ions (3,898 cations and 1,410 anions) were used to identify the compounds in volatile oil. GC-MS hyphenated techniques were used to obtain the volatile oil GC-MS total ion flow chart, by computer library search, with the peak of the mass sliver map data and matching with the literature and then check, determine the most of the chemical composition of volatile oil in fruit and seed of *C. spinosa*.

### Statistical analysis

The complete dataset including all replicates was used for cluster analysis. Both type of analysis, the ratio of the signal relative to that of the average in the four kinds of materials were log_2_ transformed, according to previously described methods [44]. PermuMatrix Version 1.9.3 EN was used for the hierarchical cluster analysis, with the distance measures based on Pearson's correlations, and the data for the correlation matrix was represented as a heatmap [45].
